# A Comprehensive Review of AIM2 in Cancer: Functional Roles, Molecular Pathways, and Clinical Relevance

**DOI:** 10.1155/jimr/9940636

**Published:** 2026-02-12

**Authors:** Nuo Wang, Shaoxian Wu, Zhang Fang, Lujun Chen, Xiao Zheng

**Affiliations:** ^1^ Department of Tumor Biological Treatment, The Third Affiliated Hospital of Soochow University, Changzhou, Jiangsu, China, suda.edu.cn; ^2^ Jiangsu Engineering Research Center for Tumor Immunotherapy, Changzhou, Jiangsu, China; ^3^ Institute for Cell Therapy of Soochow University, Changzhou, Jiangsu, China

**Keywords:** absent in melanoma 2, biological mechanisms, cancer, immunotherapy, pyroptosis

## Abstract

Inflammation has been recognized as a component of cancer progression, and inflammasome receptor components also regulate various biological processes, including cell proliferation, gene transcription, and tumorigenesis. Accumulating evidence indicates that the AIM2 inflammasome influences tumor progression via multiple concurrent mechanisms, one of which involves the negative regulation of STING‐dependent type I interferon signaling. Consequently, AIM2 may be a potential molecular target for cancer immunotherapy, and further studies are expected to reveal its application prospects in cancer therapy. Extensive research has been published on AIM2 in colorectal cancer, although it is known to also function in many other cancers. This review summarizes the diverse modes through which AIM2 in different cancers and discusses the possible future clinical application of AIM2. This review aims to provide more evidence for better methods of cancer treatment.

## 1. Introduction

Cancer remains one of the leading contributors to global mortality, with its overall burden projected to rise substantially over the coming decades [1]. A multifaceted approach in public health is needed to reduce the disease burden of cancer, and the key aspects include prevention and risk reduction, early detection and screening, access to quality care, environmental and occupational health, research and innovation, and community engagement and support [2]. Emerging areas in the research to combat cancer include precision medicine [3], immunotherapy [4], liquid and synthetic biopsies [5], artificial intelligence and machine learning [6], et cetera.

Disruption of cellular homeostasis and persistent inflammatory signaling are increasingly recognized as fundamental drivers of tumor development [7]. Within this framework, inflammasomes and autophagy function as interconnected regulatory systems that integrate stress sensing with immune and metabolic adaptation, thereby shaping tumor initiation, progression, and responses to therapy. Autophagy does not exert uniform effects in cancer; instead, it plays context‐dependent roles that may restrain malignant transformation by limiting cellular damage and promoting regulated cell death [8]. In cancer, autophagy does not exert uniform effects; rather, it can limit malignant transformation by restricting cellular damage and promoting regulated cell death [9]. Conversely, under distinct inflammatory conditions, autophagy may facilitate tumor progression by sustaining inflammation‐associated programs and adaptive survival mechanisms [10]. This context‐dependent functional duality highlights the complexity of inflammatory–autophagic regulation within the tumor microenvironment and underscores the importance of identifying upstream sensors, such as AIM2, that dictate whether these pathways converge toward tumor suppression or tumor promotion [11].

Inflammasome–autophagy interactions represent an important regulatory axis in inflammatory signaling and cancer biology [12]. Rather than functioning independently, inflammasome activation and autophagic pathways intersect to coordinately regulate inflammatory outputs and cellular homeostasis within the tumor microenvironment [13]. In multiple cancer‐related settings, autophagy has been reported to limit excessive inflammasome activity by reducing inflammatory stress, including responses associated with pro‐inflammatory cytokines such as IL‐1 family members and related downstream signaling cascades [14]. Conversely, inflammasome activation and the resulting cytokine milieu, including mediators, such as IL‐1 and IFN‐γ, can influence autophagic flux under conditions of cellular damage and metabolic stress, underscoring a bidirectional regulatory relationship between these pathways and highlighting the importance of upstream regulators, such as AIM2, that link cytosolic danger sensing with inflammatory and autophagic control [15, 16]. Such sensor diversity confers robustness and adaptability to inflammasome signaling [17].

Induced by type 1 interferons, AIM2 is a member of the PYHIN protein family (also known as the HIN‐200 family), which comprises proteins that contain both pyrin and HIN domains [18]. It is distinct from the other inflammasomes reviewed above in that it does not sense pathogen‐associated molecular patterns, damage‐associated cues, or metabolic disturbances. Instead, it binds cytosolic double‐stranded DNA (dsDNA) through a distinct recognition mechanism that triggers inflammasome assembly via the recruitment of ASC and caspase‐1 [19]. This activation leads to the secretion of bioactive IL‐1β and IL‐18 and the induction of pyroptotic inflammatory cell death, effectively coupling cytosolic DNA (often resulting from genomic instability) to an inflammatory response [20]. Cytokine release and pyroptosis are both essential for fighting infections and for the immune system to detect and suppress tumors. Across multiple cancer types, particularly colorectal cancer, AIM2 has been shown to exert tumor‐suppressive functions by restraining cellular proliferation and promoting pyroptosis, which sets it apart from the broadly responsive NLR‐based inflammasomes. However, in certain situations, AIM2 may also promote tumor progression through inflammasome‐independent mechanisms and immune modulation, which underscores its complex, context‐dependent role in tumor biology.

In this work, we discuss the current and future directions of targeting AIM2 inflammasomes in cancer therapy by reviewing the structure and activation pathways of AIM2, its role in different cancers, and its complex regulatory mechanisms. Various preclinical investigations are underway to harness the activity of AIM2 for therapeutic benefit, and a detailed understanding of AIM2 and its regulation can help develop novel therapies that leverage inflammasome activation and reveal new drug targets.

## 2. The Workings of AIM2

The unique ability of AIM2 was identified in the early discoveries of innate immunity [21]. The HIN domain of AIM2 comprises two oligonucleotide/oligosaccharide‐binding (OB) folds that cooperatively bind dsDNA, with each OB fold interacting with one DNA strand. AIM2 engages dsDNA mainly through electrostatic interactions between positively charged residues within its OB folds and the negatively charged DNA phosphate backbone, resulting in sequence‐independent binding due to the lack of direct base‐specific recognition by the HIN domain. However, efficient AIM2 inflammasome activation requires a minimum dsDNA length of approximately 70 base pairs, and the activation is optimal when the dsDNA is at least 200 base pairs in length [22–24].

AIM2 is primarily expressed in immune cells but is also present in certain tumors and non‐immune cells, where its expression increases in response to bacterial or viral infections and inflammatory stimuli and is regulated by transcription factors such as NF‐κB and TNF‐α [25]. In models of experimental autoimmune encephalomyelitis (EAE) and T cell–driven colitis, AIM2 displays dual immunoregulatory roles, acting as a canonical inflammasome sensor that shapes innate and epithelial responses while independently modulating regulatory T cell (Treg) function through a T cell–intrinsic, inflammasome‐independent mechanism [26]. Many studies indicate that AIM2 deficiency in cancer models is associated with increased cell migration, pseudopodia formation, wound healing, and metastasis, and that reintroducing AIM2 can reduce these effects. However, the findings are context‐dependent and may not apply uniformly to all tissues or tumor types.

## 3. The Roles of AIM2 in Cancers

### 3.1. Colorectal Cancer

In colorectal cancer, AIM2 exerts tumor‐suppressive effects through the recognition of cytoplasmic dsDNA, which triggers inflammasome assembly and subsequent caspase‐1 activation [27, 28]. Activated caspase‐1 processes gasdermin D to liberate its pore‐forming N‐terminal fragment, which oligomerizes within the plasma membrane and induces pyroptotic cell death [29].

Through the activation of caspase‐1, AIM2 also triggers the release of IL‐1β and IL‐18. IL‐18 increases the availability of IL‐22 by decreasing the level of IL‐22 binding protein, and the abundance of IL‐22 promotes the expression of antimicrobial peptides (e.g., Reg3β and Reg3γ) that help maintain gut microbiota balance and lower colorectal cancer risk [30]. Under normal conditions, AIM2 upregulates PTEN, a tumor suppressor enzyme whose gene is located on chromosome 10, and interacts with DNA‐PK in colonic epithelial cells to inhibit the phosphorylation of AKT and c‐Myc, thus preventing colon tumor formation.

#### 3.1.1. Prognosis of Patients and Expression in Tumor Cells

Mutations in the *AIM2* gene are linked to colorectal cancer, and decreased *AIM2* expression is correlated with higher mortality rates in humans [31, 32]. Dihlmann et al. [33] investigated the correlation between AIM2 in tumor cells and patient prognosis over a 5‐year follow‐up period and indicated that poor outcome in colorectal cancer is intimately related to the lack of AIM2 in tumor cells. Zhang et al. [34] collected 142 colorectal cancer tissue specimens and found that the median survival time was 48 months for the patients with low AIM2 levels but 61 months for those with high AIM2 levels. These findings suggested that AIM2 expression is closely linked to the prognosis of colorectal cancer patients.

#### 3.1.2. Inhibition of Stem Cell Proliferation

Intestinal stem cells have been defined as the “origin cells” of bowel cancer [35, 36]. Proliferating cells in the colons of WT and AIM2^−/−^ mice can be identified by BrdU and Ki67 staining. Man et al. [37] injected mice with azoxymethane and found increased colorectal tumorigenesis in AIM2^−/−^ mice, as evidenced by a higher tumor burden and elevated numbers of BrdU^+^ and Ki67^+^ cells. Their results indicated that AIM2 functions as a tumor suppressor by limiting excessive cell proliferation and modulating inflammatory responses, thereby mitigating the carcinogenic effects of azoxymethane. Patsos et al. [38] found that the restoration of AIM2 in AIM2‐deficient colon cancer cells suppressed cell proliferation and viability, leading to G2/M cell cycle arrest. Noticing that AIM2 restoration affected cell adhesion and invasion, upregulated genes like *VIM* and *MCAM*, and downregulated *ANXA10* and *CDH1*, they concluded that AIM2 served to reduce cell proliferation but promote an invasive phenotype in colon cancer cells.

#### 3.1.3. Maintenance of Microbiota Homeostasis

Assessing and modulating the patient microbiome is increasingly viewed as a key component of precision medicine. Altering the gut microbiome may enhance the efficacy of various cancer treatments as the microbiome shapes cancer development, modulates immune surveillance, and affects therapeutic response [39–41]. Inflammasome signaling limits intestinal inflammation and the development of colitis‐associated colon cancer through caspase‐1 activation and subsequent secretion of IL‐1β and IL‐18. Moreover, chemotherapy‐induced damage to the gastrointestinal tract releases significant amounts of DNA, which is packaged into exosomes and taken up by immune cells, thereby driving AIM2 inflammasome activation and amplifying the release of IL‐1β and IL‐18 [28, 42].

AIM2 is essential for maintaining gut microecological balance and preventing intestinal inflammation by regulating the IL‐18/IL‐22BP/IL‐22 axis, STAT3 signaling, and the expression of selective antimicrobial peptides in the intestinal microbiota [30, 43]. Ratsimandresy et al. [30] showed that the AIM2 inflammasome is essential for maintaining intestinal homeostasis and preventing colitis through the IL‐18/IL‐22/STAT3 pathway. They found that in intestinal epithelial cells, AIM2‐mediated IL‐18 production decreases the expression of IL‐22 binding protein (IL‐22BP), thereby increasing the IL‐22 level, which in turn stimulates antimicrobial peptide expression (e.g., Reg3β and Reg3γ) to help mitigate colitis. AIM2‐deficient mice harbor a dysbiotic gut microbiome that exacerbates tumor development in colorectal cancer, and 16S rRNA sequencing of their fecal matter shows elevated abundance of Akkermansia and depletion of Prevotellaceae, both of which are bacterial populations previously linked to colon tumorigenesis [37, 44, 45]. Nevertheless, the microbial balance can be restored, and the tumor burden can be reduced significantly, through co‐feeding healthy wild‐type and AIM2‐deficient mice to let them exchange their gut microbiota.

#### 3.1.4. Regulation of Signaling Pathways

Aberrant activation of the PI3K/Akt/mTOR pathway is widely observed in solid malignancies, with its core components commonly dysregulated across multiple cancer types [46–48]. In this pathway, Akt is phosphorylated at threonine 308 (Thr 308) by 3‐phosphoinositide‐dependent kinase 1, while serine 473 (Ser 473) is phosphorylated by the mTOR–Rictor complex. In colorectal tumors, mutations in the PI3K‐Akt pathway are common, and many studies have focused on finding additional molecular factors that influence the PI3K/Akt/mTOR pathway [49–52]. Tumors can enhance growth not only through genetic mutations but also by altering the mRNA and protein expression levels in key signaling pathways. For instance, the elevated expression of Rictor (an essential component of mTORC2) has been documented across multiple malignancies (including those of the brain, lung, breast, stomach, liver, and colon) [53–56]. Akt contributes to tumor development through its regulation of cell cycle dynamics, cell death programs, and cell motility. It plays a crucial role in regulating cell survival, angiogenesis, insulin signaling, and tumor development by preventing apoptosis through the phosphorylation of pro‐apoptotic proteins such as Bad and FOXO3a and by activating oncogenic proteins like MDM2, IKKα, and Skp2 [57–59]. It phosphorylates and inactivates TSC2, which normally suppresses mTORC1, leading to enhanced cellular growth and protein synthesis through mTOR pathway activation [60, 61].

AIM2 can both inhibit and indirectly promote Akt activation depending on the cellular context and environmental cues. Its regulatory effects on Akt are nuanced and serve as part of a complex network that balances cell proliferation, apoptosis, and immune responses. In intestinal epithelial cells, AIM2 activates Akt to enhance the expression of tight junction proteins, thus playing a critical role in preserving the integrity of the epithelial barrier [62]. In colorectal tumors, AIM2 interacts with and functionally inhibits DNA‐PK, which leads to reduced Akt phosphorylation and, consequently, diminished tumor cell proliferation [63]. It has been noted that DNA‐PK has the capability to phosphorylate Akt at Ser 473 directly or indirectly [64–66], and the expression and activity of DNA‐PK are elevated in colorectal tumors [67–69]. In the HCT‐116 human colon cancer cell line, restoring the AIM2 function reduces the Akt activation levels [70, 71]. In organoid cultures grown in a stromal gel matrix with appropriate growth factors, AIM2‐deficient colonic epithelial cells show both an elevated baseline and an increased IGF‐1‐stimulated level of phosphorylated Akt, indicating that AIM2 normally restrains Akt activity in these cells [72]. However, while AIM2 plays a significant role in the innate immune response, its direct effect on Akt activation within macrophages appears to be minimal or not well‐characterized [73, 74].

**Figure 1:**
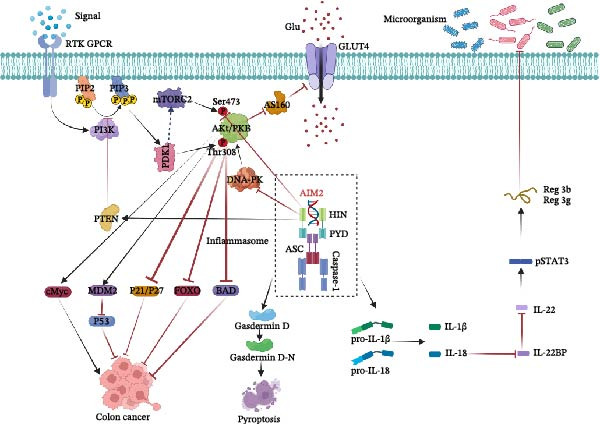
AIM2 in colorectal cancer. Caspase‐1, cysteine‐requiring aspartate protease; DNA‐PK, DNA‐dependent protein kinase; IL‐22BP, IL‐22 binding protein; mTORC2, mTOR complex 2; PDK1, 3‐phosphoinositide‐dependent protein kinase 1; PI3K, phosphatidylinositol 3‐kinase; pSTAT3, phosphorylated signal transducer and activator of transcription 3. P53, tumor suppressor protein encoded by *TP53*; PIP2, phosphatidylinositol 4,5‐bisphosphate; PTEN, phosphatase and tensin homolog deleted on chromosome ten10; Reg 3g, recombinant regenerating islet derived protein 3.

AIM2 may also regulate Akt through non‐inflammasome pathways. For example, in a colorectal cancer model, AIM2‐deficiency is associated with elevated Ser473 p‐Akt and decreased PTEN. PTEN dephosphorylates PIP3 at the cell membrane, thereby counteracting the pro‐proliferative PI3K/Akt signaling pathway. Studies have shown that SIRT6 inhibits colon cancer by increasing PTEN and PIP2 expression, stabilizing PTEN, and reducing its ubiquitination, which further suppresses PI3K/Akt signaling. In addition, by interacting with DNA‐PK, AIM2 restricts the ability of DNA‐PK to phosphorylate and activate Akt. Appropriate levels of DNA‐PKcs are necessary for genome stability, and the excessive expression of DNA‐PKcs can contribute to cellular proliferation and potentially lead to carcinogenesis by stabilizing the c‐Myc oncoprotein, primarily through the Akt/GSK‐3 pathway [37, 74]. Wilson et al. [28] revealed that AIM2 interacts with DNA‐PK to limit Akt activation, thereby preventing the formation of polyps, hyperplasia, and dysplasia, and suggested that Akt inhibitors can be a potential therapeutic strategy for AIM2‐deficient cancers. AIM2 does not appear to be associated with the MAPK pathway [75] or the NF‐κB family of transcription factors [76], although both are key to cell survival, proliferation, apoptosis. Figure 1 summarizes the mechanisms through which AIM2 regulates colorectal cancer.

### 3.2. Gastric Cancer (GC)

AIM2 plays complex and context‐dependent roles in GC, acting as both a tumor suppressor and a tumor promoter through distinct inflammasome‐independent and ‐dependent mechanisms.

#### 3.2.1. Inflammasome‐Independent Tumor‐Suppressive Functions

Clinical and experimental evidence indicates that AIM2 serves as an independent prognostic indicator in GC. Patients with low AIM2 expression exhibit poorer survival outcomes, whereas higher AIM2 levels correlate with prolonged survival and reduced tumor aggressiveness. Mechanistically, AIM2 limits the proliferative and migratory capacity of GC cells through an Akt‐dependent mechanism [77]. In resting cells, AIM2 interacts with DNA‐PK to restrain its activation [28]. The absence of AIM2 leads to DNA‐PK–mediated hyperactivation of Akt, thereby enhancing GC cell proliferation and motility.

Long‐term chronic inflammation is significantly correlated with gastrointestinal tumors and GC [78–80], and inflammatory factors are also key mediators through which AIM2 regulates the initiation and progression of GC [81, 82]. Intestinal metaplasia and pseudopyloric metaplasia, both direct consequences of persistent gastric inflammation, have been recognized as early indicators of gastric adenocarcinoma in humans [83]. Bioinformatic analysis of TCGA data further reveals that AIM2 mRNA expression is significantly elevated in GC tissues relative to normal gastric mucosa and that its level strongly correlates with overall survival [84]. In addition, both in vitro and in vivo experiments show that upregulation of CCL19 inhibits GC cell proliferation and tumor growth by activating the CCR7/AIM2 signaling pathway [85]. These results highlight a tumor‐suppressive role of AIM2 that operates independently of inflammasome activation, primarily through modulation of the DNA‐PK/Akt axis and immune‐related signaling.

#### 3.2.2. Inflammasome‐Dependent Tumor‐Promoting Effects

Conversely, AIM2 can promote gastric tumorigenesis through inflammasome‐dependent inflammation, particularly during *Helicobacter pylori* infection. Dawson et al. [86] demonstrated that AIM2‐deficient mice exhibited less gastric tissue damage, reduced inflammation, and lower epithelial proliferation following *H. pylori* infection compared with wild‐type controls. The presence of AIM2 exacerbated gastric lesions and glandular hyperplasia, suggesting that the AIM2 inflammasome drives infection‐associated gastric pathology.

Mechanistically, AIM2 physically interacts with EB1 to enhance epithelial cell migration and tumorigenesis, and overexpression of AIM2 increases tumor burden in GC xenografts. Elevated AIM2 and EB1 expression in gastric epithelium is independently associated with poor patient prognosis [87]. Downstream cytokines of AIM2 inflammasome activation, including IL‐18 and IL‐1β, further amplify tumor‐promoting inflammation. IL‐18 enhances the proliferative potential of GC cells through the ASC/IL‐18/NF‐κB signaling axis [19, 88, 89], while IL‐1β overexpression induces inflammation‐associated carcinogenesis by recruiting myeloid‐derived suppressor cells (MDSCs) [90].

#### 3.2.3. Therapeutic Implications

Although small‐molecule inhibitors targeting inflammasome components (e.g., NLRP3, NLRP1, NLRC4, and AIM2) have been developed [90], they are in vivo specificity and anticancer efficacy remain uncertain due to tissue‐ and cell type–specific roles of inflammasome complexes. In GC, therapeutic strategies may depend on identifying disease‐relevant PRRs. Given the lack of validated ASC inhibitors, IL‐18–neutralizing therapies may provide a promising approach against inflammasome‐driven GC progression.

These opposing effects underscore the complexity of AIM2 signaling in gastric carcinogenesis. Further studies are warranted to delineate the temporal and molecular conditions under which AIM2 transitions between its protective and pathogenic roles, which will be critical for developing AIM2‐targeted therapeutic strategies in GC.

### 3.3. Hepatocellular Carcinoma

In HCC, AIM2 displays a highly context‐dependent role, with its function influenced by disease stage, cell type, and microenvironmental factors such as HBV infection and inflammation [91, 92]. Evidence from early‐stage HCC is mixed: while some studies suggest that AIM2 restrains excessive inflammatory responses by modulating Kupffer cell inflammasome activity [93], others report that AIM2 inactivation can paradoxically reduce early liver damage and tumor formation [94]. This apparent contradiction highlights the complexity of AIM2 function and suggests that its role cannot be generalized across disease stages or cell types. It underscores the importance of designing studies that carefully consider temporal and cellular context.

In advanced HCC, AIM2 functions predominantly as a tumor suppressor, and its downregulation is associated with increased tumor aggressiveness, metastasis, and poorer prognosis [95–97]. At the mechanistic level, AIM2 operates through both inflammasome‐dependent signaling—characterized by caspase‐1 activation and IL‐1β/IL‐18 production—and inflammasome‐independent routes involving mTOR inhibition, autophagy regulation, and modulation of the p53 pathway [98–100]. This dual functionality likely explains why the same molecule can exhibit both protective and potentially pro‐tumorigenic roles depending on the cellular and microenvironmental context. From a subjective standpoint, this complexity represents both a challenge and an opportunity: therapeutic strategies targeting AIM2 must account for these divergent mechanisms to avoid unintended effects.

In HBV‐related HCC, AIM2 is frequently downregulated due to virus‐mediated immune evasion. HBx promotes AIM2 degradation via ubiquitination, and HBeAg suppresses AIM2 inflammasome activity in peripheral blood mononuclear cells [98, 101], collectively weakening host immune clearance and promoting persistent viral infection and tumor progression [95]. This clearly positions AIM2 not only as a tumor suppressor but also as a key mediator of host‐viral interactions, emphasizing its potential relevance in HBV‐endemic regions. The emerging evidence of crosstalk between AIM2 and nuclear DNA sensors like IFI16, which can activate p53 signaling, further indicates a sophisticated regulatory network linking cytoplasmic and nuclear DNA responses [99, 100]. This area remains underexplored and warrants targeted investigation.

Preclinical studies confirm that activating AIM2 in HCC cells triggers inflammatory and cell death pathways, significantly reducing tumor growth in xenograft models [102, 103]. Subjectively, these findings suggest that AIM2 represents a promising therapeutic target, particularly for strategies aimed at combining immune modulation with metabolic regulation. Clinically, AIM2 expression could serve as a prognostic biomarker for metastasis and recurrence, and restoring its function may offer therapeutic benefit.

Despite these insights, the field remains in its infancy. Most studies are preclinical, sample sizes are limited, and the context‐dependent effects of AIM2 across disease stages, cell types, and viral status are not fully delineated. From my perspective, future research should prioritize: (1) systematic investigation of AIM2 function across early versus late HCC; (2) integration of inflammasome‐dependent and independent mechanisms; (3) exploration of viral modulation of AIM2 signaling; and (4) clinical validation of AIM2‐targeted interventions. Taken together, AIM2 represents a complex but highly promising target in HCC, with potential implications for personalized therapy, prognostic assessment, and the development of combination strategies that leverage both immune and metabolic pathways.

### 3.4. Lung Cancer

Non–small cell lung cancer (NSCLC) represents the predominant histological subtype of lung cancer, comprising roughly 85% of cases. In contrast to its tumor‐suppressive role reported in other contexts, AIM2 has been implicated in promoting tumor growth in NSCLC [104]. Zhang and Liu [105] reported that the AIM2 intronic variant rs1103577 is significantly associated with an increased risk of NSCLC, particularly among smokers, suggesting that genetic variations in AIM2 may contribute to NSCLC susceptibility.

#### 3.4.1. Inflammasome‐Dependent Mechanisms

Zhang et al. [106] illustrated that the AIM2 inflammasome plays a pro‐tumorigenic role in NSCLC. While inflammasome activation and IL‐18 signaling exert protective effects in colorectal cancer, excessive inflammation mediated by IL‐1 signaling can promote metastasis in lung cancer [107, 108]. AIM2 activation induces ASC speck formation and enhances the maturation of IL‐1β [97]. As a recognized pro‐tumor factor in NSCLC, IL‐1β promotes cell proliferation and migration through the COX‐2/HIF‐1α pathway [109, 110].

Colarusso et al. [111] demonstrated that cigaret smoke drives AIM2 inflammasome activation and promotes the accumulation of immunosuppressive cell populations—including dendritic cells, Arg I^+^ macrophages, MDSCs, and Tregs—and the upregulation of inflammatory mediators (IL‐1α, IL‐1β, IL‐33, TNFα, IL‐10, and TGF‐β) through mechanisms independent of caspase‐1 and STING. Similarly, Sorrentino et al. [112] reported that in human lung cancer, tumor‐associated plasmacytoid dendritic cells (pDCs) engage the AIM2 inflammasome to shape an immunosuppressive tumor microenvironment that supports tumor growth [113].

Furthermore, Zhang et al. [106] found that AIM2 overexpression in NSCLC cells promotes proliferation and migration, whereas AIM2 knockdown leads to arrest at the G2/M phase of the cell cycle accompanied by reduced cell viability. These oncogenic effects depend on inflammasome assembly—marked by caspase‐1 activation and IL‐1β maturation—and are abolished by either the caspase‐1 inhibitor VX‐765 or ASC siRNA [106]. Yu et al. [114] showed that the natural flavonoid luteolin exerts antitumor effects in NSCLC by downregulating AIM2 expression and suppressing AIM2 inflammasome activation. Together, these findings highlight that AIM2’s tumor‐promoting role in NSCLC is largely mediated through inflammasome‐dependent IL‐1 signaling.

#### 3.4.2. Inflammasome‐Independent Mechanisms

AIM2 can also promote NSCLC development via inflammasome‐independent pathways. Qi et al. [115] demonstrated that AIM2 enhances NSCLC progression by modulating mitochondrial dynamics—reducing mitochondrial fusion, increasing ROS production, and activating the MAPK/ERK pathway independently of inflammasome formation. Traughber et al. [116] found that while myeloid cell–specific gasdermin D drives lung cancer metastasis through inflammasome activation and IL‐1β release, lung cancer cells maintain basal AIM2 expression but lack canonical inflammasome components, such as NLRP3, suggesting that AIM2 may sense cytosolic DNA in a noncanonical manner without forming a functional inflammasome. Alanazi et al. [117] reported that AIM2 promotes KRAS‐driven lung adenocarcinoma (LUAD) through activation of MAPK and STAT3 signaling, independent of inflammasome activity.

More recent studies further indicate that AIM2 modulates DNA‐PK complex formation and NF‐κB signaling to promote radioresistance and epithelial–mesenchymal transition (EMT) in NSCLC [118, 119]. Moreover, AIM2 activation in tumor‐associated fibroblasts can enhance IL‐6 and CXCL12 secretion, thereby indirectly activating STAT3‐mediated tumor‐promoting pathways [120]. These findings collectively suggest that AIM2 exerts multifaceted oncogenic functions in NSCLC through metabolic, mitochondrial, and transcriptional regulation beyond its canonical inflammasome activity.

#### 3.4.3. Immune Evasion and Clinical Significance

Among NSCLC subtypes, LUAD is the most prevalent, characterized by distinct molecular and clinical features. In LUAD, AIM2 has emerged as an independent prognostic biomarker, with high expression correlating with smoking history, wild‐type EGFR/KRAS/ALK status, and poor overall survival [120–122]. Colarusso et al. [111] further reported that elevated AIM2 expression in LUAD tissues is associated with an immunosuppressive microenvironment enriched in resting dendritic cells and Tregs. AIM2 promotes immune evasion and metastasis by upregulating PD‐L1 through NF‐κB/STAT1 and JAK/STAT3 pathways and inducing M2 macrophage polarization [122].

**Figure 2:**
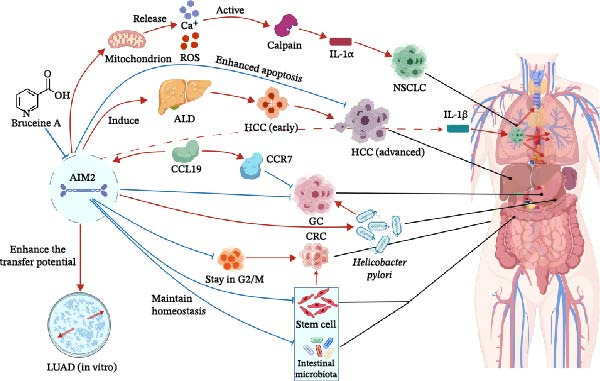
AIM2 in different kinds of cancers. ALD, alcoholic liver diseases; CCL19, chemokine (C–C motif) ligand 19; CCR7, chemokine (C–C motif) receptor 7; CRC, colorectal cancer; GC, gastric cancer; HCC, hepatocellular carcinoma; LUAD, lung adenocarcinoma; NSCLC, non‐small cell lung cancer; ROS, reactive oxygen species.

In addition, recent evidence suggests that AIM2 interacts with the STING–TBK1 axis to modulate antiviral‐like immune signaling, which could modulate patient responses to PD‐1/PD‐L1 blockade [123]. Collectively, these findings demonstrate that AIM2 contributes to NSCLC progression through both inflammasome‐dependent and ‐independent mechanisms, promoting immune suppression and metastasis. Targeting AIM2 or its downstream effectors may represent a promising strategy for improving therapeutic outcomes in LUAD. However, AIM2’s pro‐tumorigenic roles are multifaceted and not fully understood. Future studies should validate its prognostic and immunotherapy predictive value in large, multicenter cohorts, map its cell type–specific functions in the tumor microenvironment using single‐cell and spatial omics, and explore strategies that simultaneously target inflammasome‐dependent and ‐independent pathways, potentially in combination with immune checkpoint blockade, to more effectively counteract AIM2‐driven oncogenesis in NSCLC. The diverse roles of AIM2 in different cancer types are summarized in Figure 2. A summary of the biological effects of AIM2 across different cancer types is provided in Table 1.

**Table 1:** Biological effects of AIM2 in different cancers.

Cancer type	Observations and/or mechanism of action	References
Colorectal cancer	Reduces the risk of cancer by inhibiting the proliferation of intestinal stem cells	[35–37]
	Upregulates downstream antimicrobial peptides to maintain intestinal microbiota homeostasis and reduce tumor incidence	[30, 37, 45]
	Triggers G2/M phase cell cycle arrest, thereby inhibiting colon cancer cell proliferation	[38]
	Inhibits the AKT pathway to regulate cell metabolism, proliferation, and apoptosis, to reduce tumorigenesis	[70, 71]
Gastric cancer	Inhibits gastric cancer cell proliferation and migration through an AKT‐dependent pathway	[77, 81, 82]
	Patients with elevated AIM2 expression have longer survival	[77, 84]
	Lack of AIM2 leads to worsening gastric immunopathology, aggravated SPEM, and enhanced apex cell atrophy	[84]
	Overexpression of CCL19 markedly inhibits gastric cancer cell proliferation and tumor growth through activation of the CCR7/AIM2 pathway, as demonstrated in vitro and in vivo	[85]
	Promotes inflammation in *Helicobacter pylori* infection by activating inflammasome and enhancing caspase‐1 cleavage and IL‐1β release, thus promoting immune cell infiltration as well as gastric epithelial cell proliferation and apoptosis	[86]
Hepatocellular carcinoma	Bruceine A alleviates ALD by inhibiting AIM2 and reduces cancer risk	[94]
	The absence of AIM2 enhances inflammasome activation, liver inflammation, and cell proliferation during the early stages of HCC	[93]
	Significantly enhances apoptosis in HCC cells and suppresses their migration	[95, 96]
Lung cancer	Promotes the growth and proliferation of NSCLC	[107]
	Acts as an oncogene	[104]
	Serves as a pyrogenic gene and is associated with the risk of NSCLC in the Han population	[105]
	Promotes the release of mitochondrial calcium and the production of ROS, which triggers calpain activation and increases IL‐1α levels, thereby accelerating the proliferation of lung tumor cells	[112]
	Promotes the proliferation and migration of NSCLC mediated by IL‐1β	[109, 110]
	Enhances the transfer potential of LUAD in vivo and in vitro	[120]
	Upregulates PD‐L1, a marker of poor prognosis	[122]
	Use the JAK/STAT3 pathway to facilitate the immune escape of LUAD	[122]
	AIM2 upregulation is strongly associated with smoking history and the absence of EGFR, KRAS, or ALK mutations in LUAD	[120, 122]
	Activates gasdermin D, increases lung cancer incidence and risk of death	[116]
	The involvement of AIM2 in chronic airway inflammation is associated with smoking and increases the risk of cancer	[111, 121]
	Functions as a tumor suppressor in KRAS‐driven lung carcinogenesis	[117]

## 4. AIM2‐Targeted Therapeutic Strategies and Clinical Translation in Common Cancers

AIM2 plays a central role in inflammasome‐driven inflammation and immune regulation in high‐incidence cancers such as NSCLC, colorectal cancer, and hepatocellular carcinoma. Therapeutic strategies targeting AIM2 include blockade of downstream cytokines (IL‑1β and IL‑18), caspase‑1 inhibition, and modulation of innate immune pathways such as cGAS‐STING. Preclinical and early clinical studies indicate these approaches can suppress tumor‐promoting inflammation and enhance anti‐tumor immunity. Combining AIM2 modulation with radiotherapy or immune checkpoint inhibitors may further improve efficacy. Challenges remain, including tumor heterogeneity, patient selection, and limited clinical trials. Overall, these strategies highlight AIM2’s potential as a precision oncology target, as summarized in Table 2.

**Table 2:** AIM2‐targeted therapeutic strategies in cancer.

Drug/strategy	Mechanism and relation to AIM2/inflammasome	Limitations/considerations	Translational potential/future directions
Canakinumab (IL‑1β inhibitor)	Inhibits IL‑1β, a key pro‐inflammatory cytokine released after inflammasome (including AIM2) activation; IL‐1β promotes tumor microenvironment formation and immune suppression [124]	Preventive potential: targeted or combination therapy efficacy not fully demonstrated (CANOPY series not met endpoints) [125]	Candidate to inhibit AIM2 downstream pro‐inflammatory signaling; potential for combination with immunotherapy or high‐risk population prevention
Inflammasome/IL‑1 axis blockade (general)	Blocks inflammasome (including AIM2) → IL‐1β/IL‑18 release → tumor‐promoting microenvironment [126]	Complex pathways, tumor‐type dependent; may affect normal immune defense; not widely applied clinically	General immune modulation; may be combined with immune checkpoint inhibitors or targeted drugs, with clear indication and patient stratification
mTOR pathway inhibition (e.g., rapamycin)	In HCC, AIM2 loss activates mTOR‐S6K1 pathway, promoting tumor growth; mTOR inhibition compensates for low AIM2 [96]	Limited clinical data directly linking AIM2; requires stratification for low AIM2‐expressing patients	Targeted therapy for low AIM2 patients; mTOR inhibitor combination therapy may improve precision
AIM2 restoration/activation (gene/molecular)	In BRAF‐mutant colorectal cancer, restoring AIM2 induces caspase‐1‐mediated cell death, suppressing tumor growth [127]	Preclinical/animal studies; gene therapy/activator development must consider safety and selectivity	Direct AIM2 activation; potential personalized gene/molecular therapy; may combine with immunotherapy or vaccines
Combination radiotherapy/immunotherapy + AIM2 modulation	AIM2 upregulation correlates with increased PD‐L1 expression and migration; inhibition may enhance immune therapy response [120]	Complex combination design: no clinical trial specifically targeting AIM2 inhibition; patient selection required	AIM2 inhibition as adjuvant to enhance radiotherapy/immunotherapy; suitable for early clinical exploration and patient stratification
IL‑18 neutralizing antibodies/IL‑18BP	Blocks IL‑18 released downstream of AIM2 activation; reduces inflammation‐driven tumor growth and immune suppression [128]	Preclinical stage; possible off‐target immune suppression; dosing/safety need evaluation	Could complement immune checkpoint therapy; suitable for tumors with high AIM2/IL‑18 activity.
Caspase‑1 inhibitors (e.g., VX‑765)	Inhibits caspase‐1 downstream of AIM2, preventing IL‑1β/IL‑18 maturation and proptosis‐mediated tumor‐promoting inflammation [129]	Limited clinical data; potential impact on host defense; tumor‐type specificity required	May reduce AIM2‐driven pro‐tumor inflammation; combination with immunotherapy promising
STING agonists/cGAS‐STING pathway modulation	Activates cGAS‐STING pathway; AIM2 senses cytosolic DNA and can cross‐regulate STING, influencing type I IFN response and immune activation [130]	Complex interplay with AIM2; overactivation may trigger systemic inflammation	Potential combination with AIM2‐targeted strategies to enhance anti‐tumor immunity; suitable for immunotherapy‐resistant tumors
Oncolytic viruses/DNA‐based vaccines	Cytosolic DNA from viral replication can activate AIM2; controlled AIM2 activation may enhance tumor antigen presentation and immune response [131]	Safety of AIM2‐mediated proptosis must be balanced; viral therapy limitations	May synergize with AIM2‐modulating immunotherapy; precision delivery may enhance efficacy
Combination checkpoint inhibitors + AIM2 modulators	AIM2 activation upregulates PD‐L1 and other immune checkpoints; inhibition may improve T cell‐mediated anti‐tumor immunity with anti‐PD‐1/PD‐L1 [120, 132]	Patient stratification needed; risk of immune‐related adverse events	Strategy to overcome immunotherapy resistance; suitable for personalized oncology approaches

## 5. Conclusions

**Figure 3:**
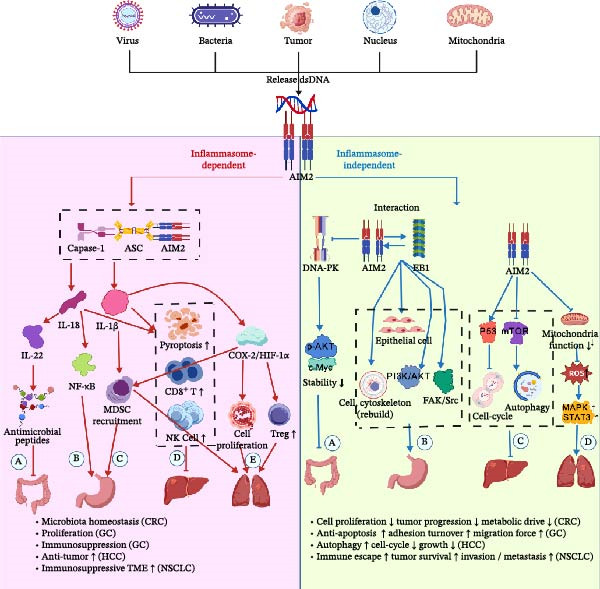
The main pathways of AIM2 inflammasome‐dependent and ‐independent functions. dsDNA released from pathogens, tumor cells, nuclei, or mitochondria activates AIM2. Left: In the inflammasome‐dependent pathway, AIM2 assembles the AIM2–ASC–caspase‐1 complex, driving IL‐1β/IL‐18 maturation, pyroptosis, NF‐κB activation, and recruitment of immunosuppressive cells, thereby influencing tumor growth and the tumor microenvironment. Right: In the inflammasome‐independent pathway, AIM2 interacts with DNA‐PK, EB1, and multiple signaling cascades (PI3K/AKT, FAK/Src, mTOR/p53, MAPK/STAT3), regulating cytoskeletal remodeling, autophagy, mitochondrial function, proliferation, invasion, and therapy resistance. Together, these pathways highlight AIM2’s context‐dependent roles across cancers.

AIM2 exerts highly context‐dependent functions across tumor types, governed by its dual roles in inflammasome‐dependent and inflammasome‐independent signaling, as summarized in Figure 3. The inflammasome‐dependent pathway involves ASC recruitment, caspase‐1 activation, and maturation of IL‐1β and IL‐18, regulating pyroptosis and innate immune responses. These effects often constrain tumor growth in colorectal and GCs, though variations in microenvironmental context can lead to divergent outcomes.

Concurrently, AIM2 engages inflammasome‐independent mechanisms, including modulation of DNA damage responses, control of proliferative and survival signaling, regulation of apoptosis, and effects on cytoskeletal dynamics and cell motility. Such pathways explain its tumor‐promoting roles in NSCLC and early hepatocarcinogenesis, while in other contexts—such as more advanced HCC or tissues where excessive inflammation is deleterious—AIM2 exhibits tumor‐suppressive activity.

Beyond functional roles, AIM2 expression serves as a molecular indicator of tumor state, often reflecting innate immune activity, cytokine programs, or DNA damage responses. Mechanistic studies manipulating AIM2 or its downstream effectors—including IL‐1 family cytokines, ASC–caspase‐1 complexes, and AIM2‐regulated metabolic or proliferative circuits—provide insight into how nucleic acid surveillance shapes tumor biology. These insights have begun to inform therapeutic development, as summarized in Table 2, although clinical translation requires careful consideration of context‐specific heterogeneity across cancer types and disease stages.

In conclusion, the balance between AIM2’s inflammasome‐dependent and independent functions determines whether it exerts tumor‐suppressive or tumor‐promoting effects, providing a framework for future mechanistic studies and the development of AIM2‐centered therapeutic strategies.

**Nomenclature**
AIM2: Absent in melanoma 2
AKT: Protein kinase B
ALD: Alcoholic liver disease
ALK: Anaplastic lymphoma kinase
AMP: Antimicrobial peptides
ASC: Apoptosis‐associated speck‐like protein containing a CARD
BA: Bruceine A
BP: Binding protein
CARD8: Caspase recruitment domain family member 8
CASP‐1: Caspase‐1
CCL19: Chemokine (C–C motif) ligand 19
CCR7: Chemokine (C–C motif) receptor 7
COPD: Chronic obstructive pulmonary disease
COX: Cyclooxygenase
CRC: Colorectal cancer
DNA‐PK: DNA‐dependent protein kinase
DSS: Dextran sodium sulfate
E3: E3 Ubiquitin ligase
EAE: Experimental autoimmune encephalomyelitis
EGFR: Epidermal growth factor receptor
EMT: Epithelial–mesenchymal transition
ERK: Extracellular signal‐regulated kinase
EZH2: Enhancer of zeste homolog 2
G2: Gap 2 phase of cell cycle
GC: Gastric cancer
GSDM: Gasdermin
GSK: Glycogen synthase kinase
HCC: Hepatocellular carcinoma
HCT‐116: Human colon tumor‐116
HIF: Hypoxia‐inducible factor
HIN: Hematopoietic interferon‐inducible nuclear proteins
IFN: Interferon
IGF: Insulin‐like growth factor
IL‐1α: Interleukin‐1α
IL‐1β: Interleukin‐1β
IL‐10: Interleukin‐10
IL‐18: Interleukin‐18
IL‐22: Interleukin‐22
IFR3: Interferon regulatory factor 3
JAK: Janus kinase
KRAS: Kirsten rat sarcoma viral oncogene homolog
LAC: Lung adenocarcinoma
LUAD: Lung adenocarcinoma
MAPK: Mitogen‐activated protein kinase
MDM2: Mouse double minute 2 homolog
MFN2: Mitofusin 2
NAIP: NLR family apoptosis inhibitory protein
NF: Nuclear factor
NLR: NOD‐like receptors
NSCLC: Non‐small cell lung cancer
OB: Oligonucleotide/oligosaccharide binding domain
P53: Tumor protein P53
PD‐L1: Programmed cell death ligand 1
PI3K: Phosphatidylinositol 3‐kinase
PIP2: Phosphatidylinositol 4,5‐bisphosphate
PIP3: Phosphatidylinositol 3,4,5‐trisphosphate
PTEN: Phosphatase and tensin homolog
RA: Rheumatoid arthritis
ROS: Reactive oxygen species
SIRT6: Sirtuin 6
SPEM: Spasmolytic polypeptide‐expressing metaplasia
STAT1: Signal transducer and activator of transcription 1
STAT3: Signal transducer and activator of transcription 3
STING: Stimulator of interferon genes
TCGA: The cancer genome atlas
TGF‐β: Transforming growth factor‐β
TNF‐α: Tumor necrosis factor‐α
TNM: Tumor‐node‐metastasis
TSC2: Tuberous sclerosis complex 2
WT: Wild‐type

## Author Contributions

**Nuo Wang:** writing – original draft, data curation, visualization. **Shaoxian Wu:** writing – review and editing, writing – original draft, funding acquisition. **Zhang Fang:** writing – review and editing, investigation, conceptualization. **Lujun Chen:** writing – review and editing, conceptualization, funding acquisition. **Xiao Zheng:** writing – review and editing, validation, supervision, resources, project administration, formal analysis, conceptualization. All the authors have contributed to funding acquisition.

## Acknowledgments

The authors declare that no Generative AI was used in the scientific writing of this manuscript.

## Funding

This work was supported by the National Natural Science Foundation of China (Grants 82303164 and 82172689), the Key R&D Project of Jiangsu Province (Grant BE2022721), the Jiangsu Funding Program for Excellent Postdoctoral Talent (Grant 2023ZB512), and the Science and Technology Support Plan (Social Development) Project of Changzhou (Grant CE20235058).

## Disclosure

All the authors have approved the manuscript for publication.

## Conflicts of Interest

The authors declare no conflicts of interest.

## Data Availability

Data sharing is not applicable to this article as no datasets were generated or analyzed during the current study.
